# Mealtime Behaviors in Dementia: A Theory-Based Understanding

**DOI:** 10.1093/geroni/igaf122.3186

**Published:** 2025-12-31

**Authors:** Zih-Ling Wang, Hilaire Thompson, Oleg Zaslavsky, Basia Belza

**Affiliations:** University of Washington School of Nursing, Seattle, Washington, United States; University of Washington, Seattle, Washington, United States; University of Washington, Seattle, Washington, United States; University of Washington, Seattle, Washington, United States

## Abstract

Many people living with dementia (PLWD) struggle with mealtimes, and these mealtime behaviors become more difficult as the disease progresses. As their condition advances, they gradually transition from eating independently to needing support from care partners. These mealtime behaviors are influenced by a complex interplay of physical and cognitive deterioration, behavioral and psychological symptoms, social dynamics, and environmental and cultural factors. The purpose of this paper is to systematically examine and compare three theoretical frameworks: (1) the Person-Environment-Occupation (PEO) Model, (2) Environmental Gerontology, and (3) the Social-Ecological Model (SEM), which enhances our understanding of these mealtime behaviors in PLWD. The PEO Model highlights the dynamic interaction between the person’s capacities, the environment, and the occupation of eating, identifying barriers and facilitators to mealtime performance. Environmental Gerontology underscores the critical role of the physical and social environment, advocating for modifications to better suit the needs and preferences of PLWD and promote well-being. The SEM provides a multilevel perspective, considering intrapersonal, interpersonal, environmental, and policy-related factors that collectively influence mealtime behaviors in the community. By examining theoretical frameworks and conceptual models, this paper aims to provide a theoretical foundation for understanding the factors influencing mealtime behaviors in PLWD. This analysis contributes to the development of strategies that enhance mealtime enjoyment, satisfaction, and nutritional well-being, thereby supporting the maintenance of eating independence across different stages of dementia.

